# Comparison of rectum fecal bacterial community of finishing bulls fed high-concentrate diets with active dry yeast and yeast culture supplementation

**DOI:** 10.5713/ab.22.0215

**Published:** 2022-09-07

**Authors:** Kai Gao, Chunyin Geng

**Affiliations:** 1 Agricultural College, Yanbian University, Yanji 133000, China; 2 Engineering Research Center of North-East Cold Region Beef Cattle Science & Technology Innovation, Ministry of Education, Yanbian University, Yanji 133000, China

**Keywords:** Active Dry Yeast, Cattle, Intestinal Bacteria, 16S rDNA, Yeast Culture

## Abstract

**Objective:**

The objective of this study was to investigate the effects of feeding active dry yeast (ADY) and yeast culture (YC) on fecal bacterial community in finishing bulls fed high-concentrate diets in the same experimental environment.

**Methods:**

Forty-five healthy finishing cattle (Simmental×Chinese Luxi yellow bulls; 24 months; 505±29 kg) were randomly divided into three groups: i) CON group (control group, only fed basal diet), ii) ADY group (fed basal diet + active dry yeast), and iii) YC group (fed basal diet + yeast culture). At the end of the trial, nine rectum fecal samples were randomly selected from each group for bacterial DNA sequencing.

**Results:**

There was no difference among groups about alpha diversity indices (all p>0.05), including ACE, Chao 1, Shannon, and Simpson indices. Principal component analysis and non-metric multidimensional scaling analysis showed a high similarity among three groups. Compared with CON group, ADY and YC groups had greater relative abundance of c_*Clostridia*, o_*Oscillospirales*, and f_*Oscillospiraceae*, but lesser relative abundance of g_*Megasphaera*, and s_*Megasphaera_elsdenii* (all p<0.01). And, the relative abundances of p_*Firmicutes* (p = 0.03), s_*Prevotella_sp* (p = 0.03), o_*Clostridiales* (p<0.01), g_*Clostridium* (p<0.01), f_*Caloramatoraceae* (p<0.01), and f_*Ruminococcaceae* (p = 0.04) were increased in the ADY group. The PICRUSt2 prediction results showed that the metabolic pathways had no significant differences among groups (p>0.05). Besides, the relative abundance of c_*Clostridia* (r = 0.42), and f_*Oscillospiraceae* (r = 0.40) were positively correlated to average daily gain of finishing bulls (p<0.05).

**Conclusion:**

Both of ADY and YC had no effect on diversity of fecal bacteria in finishing bulls, but the supplementation of ADY and YC can improve the large intestinal function in finishing bulls by increasing the abundance of cellulolytic bacteria and altering the abundance of lactic acid-utilizing bacteria.

## INTRODUCTION

The composition and function of cattle gastrointestinal microbiome plays a critical role in animal health and nutrient uptake [1]. Among them, bacteria play a dominant role in metabolomic activities of cattle [2]. To date, many studies have focused on rumen bacteria and their relationship with gastrointestinal microenvironment and production performance of cattle, but there are few studies focused on bacteria in the hindgut of beef cattle. The difference of bacterial communities between ruminant hindgut and rumen may be related to the structural and functional differences of the two digestive organs. Compared with the bacteria in rumen, the relative abundance of *Bacteroidetes* and *Spirochaetes* in the ruminant hindgut is lower, while *Firmicutes* and *Proteobacteria* is higher [3]. The hindgut microorganisms can degrade unutilized substances into volatile fatty acid to provide nutrition for the body [4], and some certain species of *Bacteroidetes* and *Firmicutes* may be related to the regulation of host behavior and intestinal immunity [5]. Therefore, the study of ruminant hindgut microorganisms is conducive to the overall understanding of ruminant gastrointestinal function.

Yeast preparations from *Saccharomyces cerevisiae* can be classified into two types according to the count of live yeast cells in products, which are active dry yeasts (ADY) and yeast culture (YC). Active dry yeasts which guarantee high number (>10^9^ colony forming units/g) of live yeast cells are sold as 100% ADY, while YC are sold as entire culture medium with a small amount of live yeast cells. Although YC contains some residual viable yeast cells, which is not a substantial source of yeast biomass, and the effective components of YC are extracellular metabolites, such as peptides, alcohols, esters, and organic acids [6]. ADY and YC have been used in ruminants to favorably modify the ruminal environment and improve production performance. Nevertheless, published literature regarding ADY or YC did not show conclusive evidence that its supplementation is beneficial for animal performance at all times [7–9]. The action mechanism of ADY and YC on growth performance of ruminants has been explored for its scientific applications and product development [10,11]. So far, most studies have paid more attention on the effects of yeast preparations on rumen microorganisms [12,13], but little on hindgut microorganisms in finishing cattle [14]. At present, it has been demonstrated that ADY can reduce fecal *Escherichia coli* O157:H7 counts [15] and alter the dominant fecal bacteria at phylum and genus levels [16]. However, the effect of YC on hindgut bacteria community and different effects of ADY and YC on the fecal bacteria community in finishing bulls fed high-concentrate diets remain unclear. Therefore, the objectives of this study were to determine the alteration of fecal bacteria community of finishing bulls fed high-concentrate diets with ADY and YC supplementation using 16S rDNA sequencing. Growth performance, carcass traits, meat quality and blood indexes were reported previously [7,17].

## MATERIALS AND METHODS

### Animal care

The study received the approval of the Animal Ethics Committee of Yanbian University (Yanji, China) and carried out in accordance with animal welfare guidelines and regulations.

### Animals, experimental design, and sample collection

The experimental design was as described in our previous study [7]. Briefly, 45 healthy finishing cattle (Simmental× Chinese Luxi yellow bulls; 24 months; with initial body weight of 505±29 kg) were randomly divided into three groups: i) CON group (control group, only fed a basal diet), ii) ADY group (fed basal diet + active dry yeasts preparation (Levucell SC, *S. cerevisiae* CNCM1-1077; white; >0.8×10^10^ CFU/g; 0.8 g/head/d), and iii) YC group (fed basal diet + yeast culture preparation (Diamond V XP, Cedar Rapids, IA, USA; 50 g/head/d). The basal diet was a total mixed ration with concentrate to forage ratio of 70:30 (as shown in Supplementary Table S1). The finishing bulls were fed twice a day at 05:00 and 17:00 for 98 days. At the end of the trial and before the morning feeding, nine cattle were randomly selected from each group for rectal fecal sampling by using sterile surgical gloves. One gram of feces was subsampled from each mixed fecal sample, snap frozen in liquid nitrogen and stored at −80°C for DNA extraction.

### Fecal bacteria DNA extraction and sequencing

Total bacterial DNA was extracted from 125 mg fecal sample using a TGuide S96 Magnetic Soil/Stool DNA Kit (Tiangen Biotech Co., Ltd, Beijing, China). DNA sequencing was performed as described previously [18]. Briefly, DNA purity and concentration were determined with a Synergy HTX multi-mode reader (Gene Company Limited, Hong Kong, China) and DNA integrity was assessed by 1.8% agarose gel electrophoresis. First-round tailed polymerase chain reaction (PCR) amplification was performed as detailed in previous research [19]. The full-length 16S rDNA sequence was amplified using the universal primers: 27F (5′-AGRGTTTGAT YNTGGCTCAG-3′) and 1492R (5′-TASGGHTACCTTGT TASGACTT-3′). The second-round tailed PCR reaction system contained the barcode primer pair (3 μL), genomic DNA (1.5 μL), nuclease-free water (10.5 μL), and KOD OneTM PCR Master Mix (15 μL). The cycling parameters were as follows: initial denaturation for 2 min at 95°C, then 98°C for 10 s, 55°C for 30 s, and 72°C for 90 s, for 25 cycles, and a final extension for 5 min at 72°C. PCR amplification products were detected by Qubit4 fluorometer (Thermo Fisher, New York, USA) and 1.8% agarose gel electrophoresis, before purification, quantification, and homogenization to create a sequence library. The marker genes were sequenced by single molecule real-time sequencing using a HiSeq 2500 system PacBio Sequel II system (Pacific Biosciences, Menlo Park, CA, USA).

### Sequence data processing and analysis

Analysis of sequence data followed a protocol described previously [19]. Effective reads were obtained by filtering the raw reads using Trimmomatic (v.0.33), identification and removal of primer sequences by cutadapt (v.1.9.1), splicing of high-quality reads by FLASH (v.1.2.7), and the identification and removal of potential chimera using the UCHIME algorithm. Operational taxonomic units (OTUs) were obtained by clustering reads at 97.0% similarity level using Usearch (v.10.0). Taxonomic annotation of OTUs based on the SILVA database (Release 132) used the naive Bayes classifier. Species abundance at phylum and genus levels was generated by QIIME2 (v.2020.6) and mapped by R (v.3.3.2). The alpha diversity indices (Chao1, ACE, Shannon, and Simpson) were evaluated using QIIME2 (v.2020.6). The alpha diversity data were analyzed using one-way analysis of variance with Dunnett T3 test. Statistical significance was set at p<0.05. Shannon curves and species accumulation curves (OTU level) were created using Mothur software and R (v.3.3.2). QIIME2 (v.2020.6) was used to determine beta diversity, the Bray Curtis algorithm to calculate the distance among samples to obtain the beta value, and three-dimensional principal component analysis (3D PCA) and non-metric multidimensional scaling analysis (NMDS) for analysis of beta diversity. Species for statistical differences among groups were analyzed using linear discriminant analysis (LDA) with LDA scores >4. Functional gene prediction analysis was carried out according to previous research [20]. Briefly, characteristic sequences were annotated using PICRUSt2 and matched with the Kyoto encyclopedia of genes and genomes database (KEGG) to predict the functional gene composition of a sample. STAMP software was used to carry out t-tests on functional abundances among groups.

### Correlation analysis

We previously reported that the ADY supplementation can significantly improve the final weight (FW), dietary dry matter intake (DMI) and average daily gain (ADG), and both supplementation of ADY and YC can significantly improve the level of serum ghrelin and serum triglyceride (TG) of finishing bulls (Supplementary Table S2, 3) [7,17]. The correlations between differential fecal bacteria and FW, DMI, ADG, serum ghrelin and serum TG were calculated by Spearman’s rank correlation analysis with p value <0.05 being considered as significant.

## RESULTS

### Diversity of the fecal bacteria in different groups of finishing bulls

**Figure 1:**
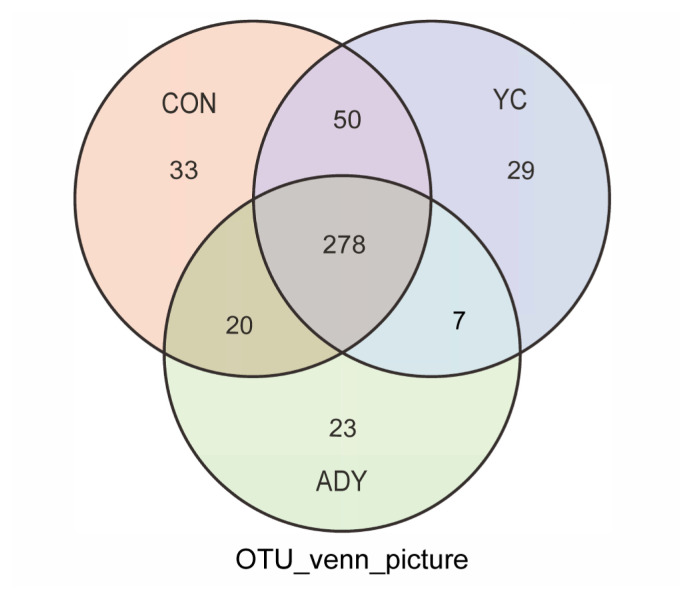
Venn diagram of number of operational taxonomic units of rectum fecal bacteria in finishing bulls. CON, control group (n = 9); ADY, active dry yeast group (n = 9); YC, yeast culture group (n = 9).

In the present study, a total of 149,858 circular consensus sequences (CCS) were obtained by identifying barcodes and average sequence efficiency (effective CCS/Raw CCS) exceeded 93% in the sequencing results from twenty-seven fecal samples in finishing bulls by sequencing the 16S rDNA genes (Supplementary Figure S1). The results of the OTUs were used to create Venn diagrams, showing the numbers of microbes and variances of different groups of finishing bulls (Figure 1). The numbers of total OTUs in CON, ADY and YC groups were 381, 328, and 364, respectively. The number of mutual OTUs from the three groups was 278, representing 63.18% of all OTUs.

**Figure 2:**
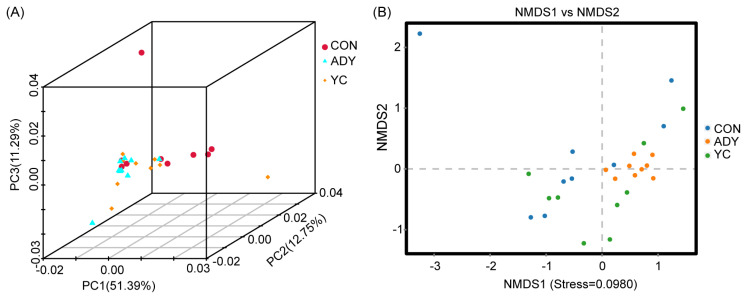
Beta diversity analysis of rectum fecal bacteria through (A) three-dimensional principal component analysis (3D-PCA) and (B) non-metric multidimensional scaling analysis (NMDS). CON, control group (n = 9); ADY, active dry yeast group (n = 9); YC, yeast culture group (n = 9).

Beta diversity was used to compare the microbial community in different samples by calculating the 3D-PCA and NMDS. As shown in Figure 2, the 3D-PCA and NMDS plots based on the Bray-Curtis distance matrix showed that the points representing fecal microorganisms in the three groups were not independent, which indicated that the community structure of large intestinal bacteria had no difference among treatments.

**Figure 3:**
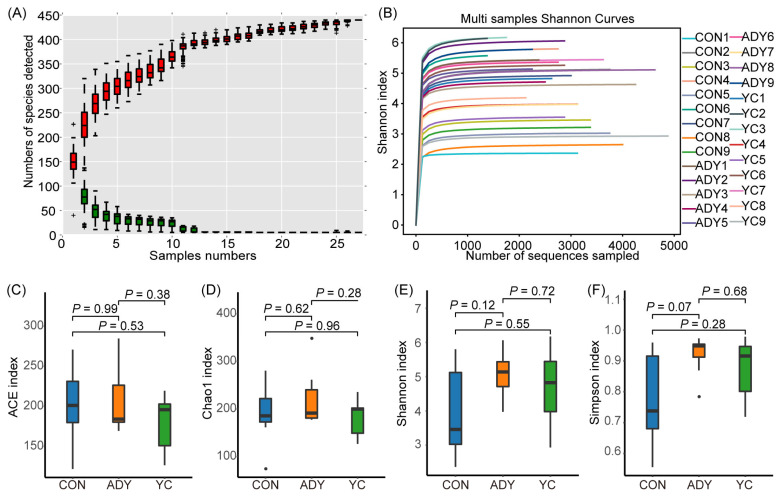
Alpha diversity of the rectum fecal bacteria in finishing bulls. (A) Species relative abundance accumulation curve, (B) the Shannon index rarefaction curves, (C) Abundance-based coverage estimator (ACE) index, (D) Chao 1 index, (E) Shannon index and (F) Simpson index. A single red box reflects the total number of species contained in the sample, and the total red box constitutes a cumulative curve. A single green box reflects the number of common species in the sample; The total green box constitutes the common quantity curve. CON, control group (n = 9); ADY, active dry yeast group (n = 9); YC, yeast culture group (n = 9).

Species relative abundance accumulation curve and the Shannon index rarefaction curves indicated that all samples provided sufficient OTU coverage to accurately describe the bacterial composition of each treatment (Figure 3A and 3B). In order to measure the diversity within the microbial community of each sample, we compared alpha diversity of the fecal bacteria in all samples by calculating the ACE, Chao1, Shannon, and Simpson indices (Figure 3C, 3D, 3E, and 3F). We found that the Simpson index in ADY group had an increased tendency compared with CON group (p = 0.07), but no significant differences were observed (Figure 3F).

### Compositional comparison of fecal bacteria in different groups of finishing bulls

**Figure 4:**
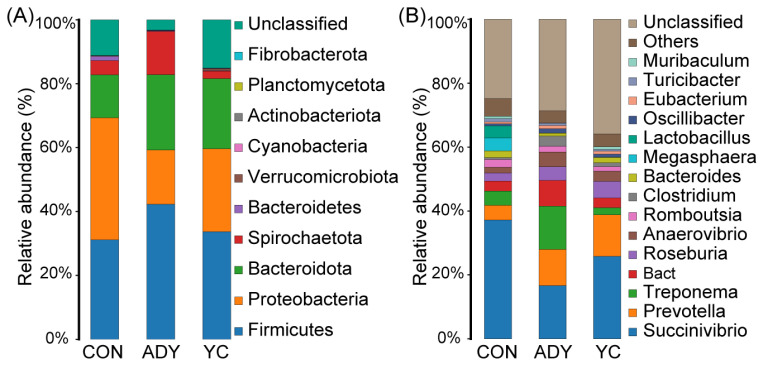
The rectum fecal bacterial community compositions at (A) phylum (all) and (B) genus (top 15) level. Taxonomy was assigned using the SILVA database version 132. The different colors of the bars represent different species, and the length of the bars represents the proportion of the species. CON, control group (n = 9); ADY, active dry yeast group (n = 9); YC, yeast culture group (n = 9).

At the phylum level, *Firmicutes* (CON vs ADY vs YC = 32.19% vs 43.18% vs 34.13%), *Proteobacteria* (CON vs ADY vs YC = 33.78% vs 15.01% vs 23.88%), and *Bacteroidota* (CON vs ADY vs YC = 15.68% vs 24.41% vs 25.76%) predominated in the hindgut of all groups, and occupied more than 80% of the microbial community (Figure 4A). The dominant bacterial genera identified in all finishing bulls’ fecal bacteria were *Succinivibrio*, *Prevotella*, *Treponema*, *Bact*, *Roseburia*, and *Anaerovibrio* (Figure 4B). Figure 4 also shows that the supplement of ADY or YC had altered abundances of specific bacterial taxa at the phylum and genus level. Hence, different types of yeast production could dissimilarly promote the growth of certain bacteria in the rectum of finishing bulls.

**Figure 5:**
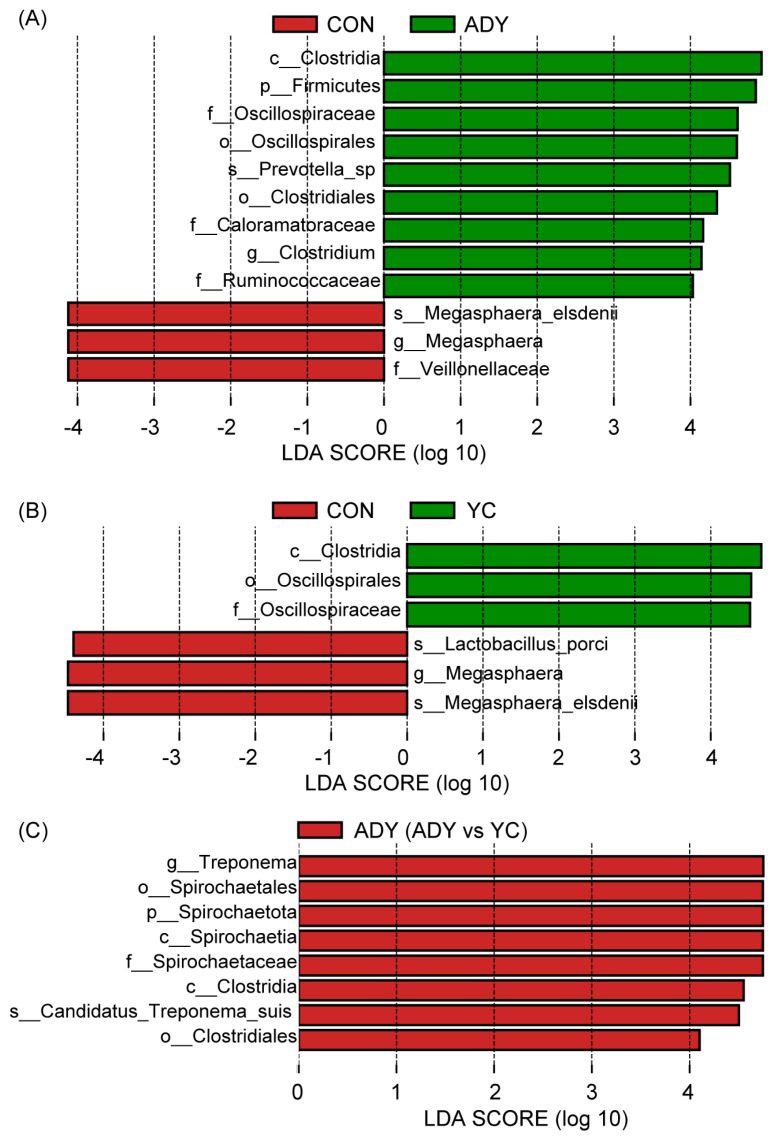
The linear discriminant analysis effect size (LEfSe) analysis of differential fecal bacteria. Linear discriminant analysis (LDA) bar showed the impact of the abundance of each species on the difference in (A) ADY vs CON, (B) YC vs CON, and (C) ADY vs YC. p-Value <0.05 and LDA score >4 were defined as significant difference. p, phylum; c, class; o, order; f, family; g, genus; s, species. CON, control group (n = 9); ADY, active dry yeast group (n = 9); YC, yeast culture group (n = 9).

Furthermore, the significantly different abundant fecal bacteria between the 2 groups were identified through LEfSe analysis and LDA. Interestingly, compared with CON group, ADY and YC groups had greater relative abundance of c_*Clostridia*, o_*Oscillospirales*, and f_*Oscillospiraceae*, but lesser relative abundance of g_*Megasphaera*, and s_*Megasphaera_elsdenii* (all p<0.01) (Figure 5A and 5B). Besides, the relative abundance of f_*Veillonellaceae* (p = 0.01) and s_*Lactobacillus_porci* (p = 0.03) were decreased in ADY and YC groups (Figure 5A and 5B), respectively. And, the relative abundances of p_*Firmicutes* (p = 0.03), s_*Prevotella_sp* (p = 0.03), o_*Clostridiales* (p<0.01), f_*Caloramatoraceae* (p<0.01), g_*Clostridium* (p<0.01), and f_*Ruminococcaceae* (p = 0.04) were increased in the ADY group (Figure 5A). Compared with the ADY group, the relative abundances of g_*Treponema* (p<0.01), o_*Spirochaetales* (p<0.01), p_*Spirochaetota* (p<0.01), c_*Spirochaetia* (p<0.01), f_*Spirochaetaceae* (p<0.01), c_*Clostridia* (p<0.01), s_*Candidatus_Treponema_suis* (p = 0.01), and o_*Clostridiales* (p = 0.02) in YC group were decreased (Figure 5C).

### Functional characterization of fecal bacteria in different groups of finishing bulls

**Figure 6:**
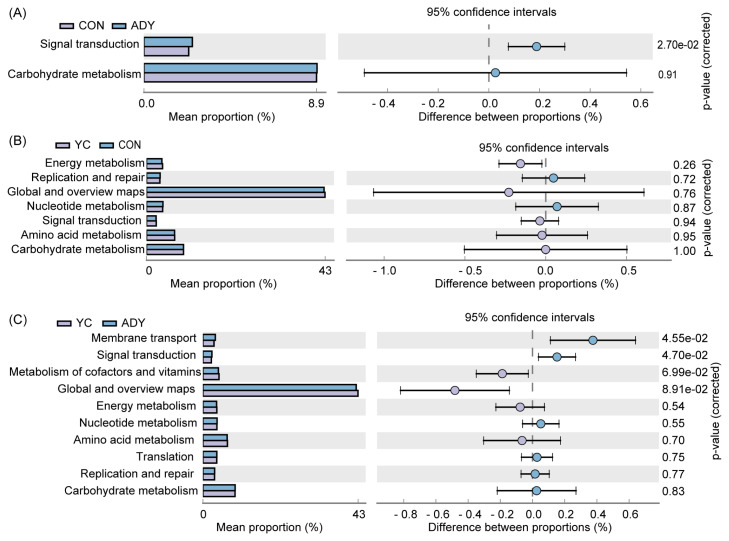
Comparison of predicted Kyoto encyclopedia of genes and genomes database (KEGG) functions of fecal bacteria at level 2 by using PICRUSt2 (top10 and p-value (corrected) <1). Functional classification of (A) ADY vs CON, (B) YC vs CON, and (C) ADY vs YC. CON, control group (n = 9); ADY, active dry yeast group (n = 9); YC, yeast culture group (n = 9).

The PICRUSt2 software was used to predict the metabolic functions of the identified bacterial 16S rDNA genes by using KEGG database. The metabolic pathways had no significant differences (p>0.05) between YC and CON groups (Figure 6B). Interestingly, the relative abundance of membrane transport and signal transduction genes were more abundant in ADY group than that in CON group (Figure 6A) or YC group (Figure 6C) (p<0.05). Besides, the functional genes of fecal bacteria in all groups were mainly associated with the following metabolic pathways: global and overviews maps, carbohydrate metabolism, amino acid metabolism, metabolism of cofactors and vitamins, nucleotide metabolism, translation, energy metabolism (in order from high to low abundance) (Supplementary Figure S2).

### Correlation analysis between differential fecal bacteria and final weight, dietary dry matter intake, average daily gain, serum ghrelin and triglyceride

**Figure 7:**
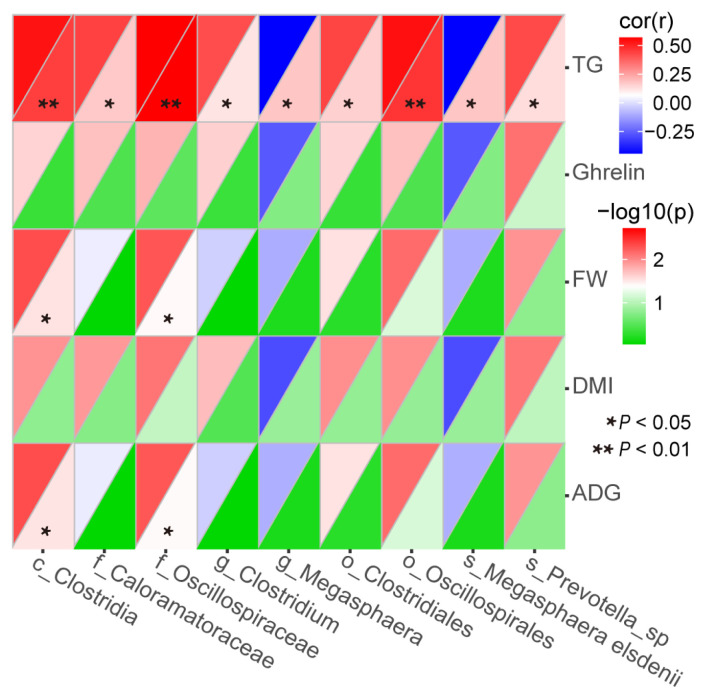
Spearman’s rank correlations between differential fecal bacteria and final weight, dietary dry matter intake, average daily gain, serum ghrelin and serum triglyceride. FW, final weight; DMI, dietary dry matter intake; ADG average daily gain; and TG, serum triglyceride. Spearman’s rank correlation coefficient (r) was from −1 to 1. r>0 and <0 represented a positive and negative correlation, respectively. The (r) value denoted the degree of correlation between variables. Only the bacteria with a relative abundance of 1%, or higher, in at least one sample were considered.

As shown in Figure 7, the relative abundance of c_*Clostridia* (r = 0.53; p<0.01), f_*Caloramatoraceae* (r = 0.44; p<0.05), f_*Oscillospiraceae* (r = 0.57; p<0.01), g_*Clostridium* (r = 0.42; p<0.05), o_*Clostridiales* (r = 0.43; p<0.05), o_*Oscillospirales* (r = 0.54; p<0.01) and s_*Prevotella_sp* (r = 0.42; p<0.05) were positively correlated with serum TG, whereas the relative abundance of g_*Megasphaera* (r = −0.44; p<0.05) and s_*Megasphaera_elsdenii* (r = −0.44; p<0.05) were negatively correlated. Meanwhile, the relative abundance of c_*Clostridia* (r = 0.42; r = 0.42), and f_*Oscillospiraceae* (r = 0.40; r = 0.40) were positively correlated with FW and ADG (all p<0.05).

## DISCUSSION

In the present study, we evaluated the impact of different types of yeast preparations (ADY and YC) on the fecal bacteria community of finishing bulls based on the high-throughput sequencing technology. Some studies reported that ADY alters the beta-diversity of rumen samples of beef steers [21]. Through the analysis of the diversity indices of samples in three groups, we found that the supplement of ADY and YC did not alter the fecal bacteria diversity in finishing bulls fed high-concentrate diets. This is consisted with Ran et al [16], who reported that neither ruminal protected nor non-protected ADY can change the structure of fecal bacteria of beef cattle. As known to us, yeast preparations can provide peptides, amino acids, vitamin and trace minerals to stimulate the growth of rumen microorganism [22]. Besides, live yeast cells of ADY can also scavenge oxygen to create an anaerobic ruminal environment for the growth and multiplication of anaerobic bacteria, which is one of the important reasons why ADY affect the structure of rumen bacterial community [23]. But a factor of scavenge oxygen by ADY supplementation may not be the key for bacteria in the ruminant anaerobic hindgut. In addition, it is also possible that almost all the nutrients of growth factors in yeast preparations are utilized in rumen microorganisms, and the contents of various nutritional factors in chyme are not different enough to cause changes in the structure of hindgut flora.

Even so, the LEfSe analysis results indicated that yeast metabolites in yeast preparations effectively regulate the relative abundance of certain fecal bacteria in the current study. For example, the supplementation of ADY and YC significantly increased the relative abundance of c_*Clostridia*, o_*Oscillospirales*, and f_*Oscillospiraceae*. As a strict anaerobe, *Clostridia* has the ability to ferment complex plant carbohydrates [24]. *Clostridia* species produce short chain fatty acids (e.g. butyrate), mucin and antimicrobial peptides to provide essential nutrients and energy and enhance epithelial barrier integrity [25]. Besides, the increase of butyrate-producing bacteria - *Clostridia* suppresses the growth of aerobic *Salmonella* by increasing butyrate concentration and decreasing epithelial oxygenation in mammalian intestine [26]. Of course, there are some potentially toxic and pathogenic strains in *Clostridium* genus [27], but beneficial strains dominate after supplementing yeast preparations in the current study, judging from the physiological state, growth performance and plasma indexes of cattle (Supplementary Table S2, S3) [7,17]. As a common gut microbiota, *Oscillospira* is a rarely cultivated bacterial genus, which can utilize glucuronate and produce all kinds of short-chain fatty acids, especially butyrate [28]. Generally, *Oscillospira* is also positively associated with fiber content of diets [29]. That means the supplementing of yeast metabolites can enhance the intestinal digestion of fiber polysaccharide in finishing bulls. It is also reported that the increase abundance of *Oscillospira* may aggravate constipation in human [30]. Hence, the addition of yeast preparation can effectively reduce the duration of calf diarrhea [31,32], which may be also related to the increased abundance of this organism. *Oscillospira* consumes glucose, ethanol, and lactic acid in the culture medium for growth and multiplication, which may be the reason why the bacteria in hindgut supplemented with yeast preparation is more conducive to adapt to high grain diet [33]. This is also confirmed by our study, in which a positive correlation was observed between c_*Clostridia*, f_*Oscillospiraceae* and serum TG, FW, ADG in finishing bulls, indicating that the relative abundance of this bacteria in the rectum increased according to the growth performance. However, the reduced relative abundance of g_*Megasphaera* and s_*Megasphaera_elsdenii* were also observed after 98 days of supplementation with ADY and YC. *Megasphaera elsdenii* belongs to family *Veillonellaceae* and is the common lactate-utilising bacteria in the rumen of grain-fed cattle [34]. the supplementation of ADY also decreased the relative abundance of f-*Veillonellaceae*. Ogunade et al [35] demonstrated that yeast preparations have the ability to increase the abundance of carbohydrate digesting bacteria and lactate-utilising bacteria. Thus, we speculate that the increased relative abundance of *Oscillospira* may compensate for the decrease of *Megasphaera* in the hindgut of finishing bulls fed high-concentrate diets. The mechanism leading to this strange phenomenon still needs further studies. Besides, the relative abundance of s_*Lactobacillus_porci* was decreased after supplementing YC. This agrees with Lesmeister et al [36], who demonstrated YC could restrain the activities of lactate-producing bacteria. With glucose as a carbon source, *Lactobacillius* can produce lactic acid [37]. Hence, reducing the production of lactic acid by decreasing the relative abundance of *s_Lactobacillus_porci* could prevent hindgut acidosis [38,39]. Our observations suggest that both ADY and YC can regulate the relative abundance of cellulolytic bacteria and lactic acid-utilizing bacteria in the hindgut and potentially improve the adaptability of the intestine to high-energy diet in finishing bulls.

Besides, the supplementation of ADY also increased the relative abundances of p_*Firmicutes*, s_*Prevotella_sp* and f_*Ruminococcaceae*. These results are consistent with Ran et al [16], who found that both ruminal protected and non-protected ADY can effectively improve the relative abundance of phylum_*Firmicutes* and genus_*Prevotella*. Irrespective of diet fed to animals, the phylum *Firmicutes* would probably be the most dominant in high grain diet [40]. Furthermore, increased relative abundance of *Firmicutes* enhanced energy harvesting in bovine and played a crucial role in increasing fat deposition in cows and feed efficiency in steers [41,42]. The polysaccharide-degrading *Prevotellaceae* bacterium has greater relative abundance in the rumen of cows fed high-concentrate diets, which can utilize and convert lactic acid into propionic acid [43]. As one of the most abundant families from the order *Clostridiales* in gut, the *Ruminococcaceae* has abundant genes encoding key carbohydrate-active enzymes, and could degrade complex plant material, including cellulose and hemicellulose, to produce short chain fatty acids (mainly acetate, butyrate, and propionate) [44]. These results suggest that ADY supplementation potentially improves the metabolism of polysaccharide in the large intestine of finishing bulls fed high-concentrate diets.

Furthermore, compared with supplementing ADY, the decreased relative abundances of o_*Clostridiales*, c_*Clostridia*, p_*Spirochaetota*, c_*Spirochaetia*, o_*Spirochaetales*, g_*Treponema*, f_*Spirochaetaceae*, and s_*Candidatus*_*Treponema*_*suis* were observed in the hindgut with supplementation of YC. As mentioned above, the great majority the *Clostridia* have a beneficial and commensal relationship with the host, although some of them are pathogenic. *Spirochaetaceae* (mainly the genus *Treponema* spp.) has the ability of fiber degradation [45,46]. Although *Treponema* include harmless commensal bacterial species, they are primarily known as potential pathogens [47]. *Candidatus Treponema suis* is known to cause colitis by invading the surface epithelium and the mucosa in pigs [48]. One species of genus *Treponema* (*T. brennaborense*), whose sequence is 90% similar to *Candidatus Treponema suis*, was found to be associated with digital dermatitis in dairy cows [49]. However, we did not find diarrhea, inflammation or other health problems in finishing bulls during the trial [7,17]. Moreover, most of researches reported that both ADY and YC also could reduce the diarrhea rate and improve immunocompetence and fecal scores of dairy calves [31,50]. Thus, the fiber degradation ability of the gut microbiota supplemented YC seems weaker than that of ADY supplementation in the current experimental condition, but it is difficult to distinguish which is more beneficial to the large intestinal health.

The fecal microbiota of beef cattle plays an important role in physiology, nutrition, health, and productivity. In the current study, phyla *Firmicutes*, *Proteobacteria*, and *Bacteroidota* predominated and occupied more than 80% of the fecal bacterial community in all finishing bulls, which is consistent with previous reports [16,51–53]. *Succinivibrio* was the first dominant bacterial group at genus level in this study. However, Ran et al [16] found *Prevotella* was the first dominant genus in fecal bacteria in cattle fed with high-grain diets. This may have been caused by different breed and dietary components. Furthermore, Hernandez-Sanabria et al [54] revealed that *Succinivibrio* were abundant in rumen of dairy cattle fed with high-concentrate and were positively correlated with feed efficiency and productivity. This indicates that the most dominant bacteria at genus level are highly variable in hindgut of finishing bulls. Although the supplementation of ADY and YC altered the relative abundance of certain fecal flora, the PICRUSt2 prediction results showed that it did not change the metabolic pathways of large intestinal bacteria, including carbohydrate metabolism, amino acid metabolism, metabolism of cofactors and vitamins, nucleotide metabolism, translation, energy metabolism. This indicates that different fecal bacteria can have similar functions in the hindgut ecosystem, and the gastrointestinal bacterial flora may achieve its stability through functional redundancy [55]. Interestingly, the relative abundance of membrane transport and signal transduction genes were more abundant in hindgut bacterial community added with ADY, which showed that feeding finishing bulls with ADY had potential to maintain the stability of intestinal flora by enhancing the response ability of intestinal bacteria to external stimuli [56].

## CONCLUSION

In conclusion, both of ADY and YC had no effect on diversity of fecal bacteria in finishing bulls, but the supplementation of ADY and YC can alter the relative abundance of some cellulolytic bacteria and lactic acid-utilizing bacteria in the hindgut, and YC had a weaker effect than ADY in the current experimental condition. Furthermore, the relative abundance of class_*Clostridia* and family_*Oscillospiraceae* were positively correlated with average daily gain of finishing bulls receiving ADY and YC. The findings contribute to a better understanding of potential effects of yeast preparations on finishing bulls fed high-concentrate diets.

## SUPPLEMENTARY MATERIAL

**Supplementary Table S1**. Ingredient and nutritional composition of basal diets (% of dry matter)

**Supplementary Table S2**. Effect of dietary supplementation of active dry yeast and yeast culture on growth performance and blood indexes of finishing bulls (n = 15)

**Supplementary Table S3**. Effect of dietary supplementation active dry yeasts (ADY) and yeast culture (YC) on blood immunoglobulin and blood hormone in finishing bulls (n = 15)

**Supplementary Figure S1**. Summary of sequence statistics of all samples

**Supplementary Figure S2**. PICRUSt2 prediction results at Class2
