# Supplementary material for: Comparison of rectum fecal bacterial community of finishing bulls fed high-concentrate diets with active dry yeast and yeast culture supplementation
Source: Anim Biosci. 2022 Sep 7;36(1):63–74. doi: 10.5713/ab.22.0215 (PMC9834660; doi:10.5713/ab.22.0215)
Supplement: Supplementary file 1 [file ab-22-0215-suppl1.pdf]

Table S1. Ingredient and nutritional composition of basal diets (% of dry matter)

Ingredient composition	Content (% of DM)	Nutritional composition[1]	Content (% of DM)
Corn silage	30.61	Dry matter, DM	71.45
Corn meal	51.14	Crude protein	12.23
Cottonseed meal	7.79	Ether extract	2.98
Soybean meal	5.79	Neutral detergent fibers	25.86
Salt	0.18	Acid detergent fibers	15.57
Sodium bicarbonate	1.04	Calcium	0.63
Compound premix[2]	3.45	Phosphorus	0.37
Total (%)	100.00	Sodium chloride	0.40
		NEg[3] (Mcal/kg DM)	1.28

[1] The value reported for nutritional composition of diets was calculated based on the nutrient analysis from ingredient samples.

[2] Supplied per kilogram of product. Ca: 160 g; P: 30 g; Cu: 450mg; Zn: 1600mg; Mn: 800 mg; I: 10mg; Co: 10mg; Se: 5 mg; vitamin A: 120 000 IU; vitamin D:55000 IU; vitamin E: 400 mg; vitamin B3: 600 mg; vitamin B5: 200 mg; monesin: 1000 mg; salt: 0.065 kg.

[3] NEg (net energy for growth) was estimated from CNCPS (6.0) values.
