# Supplementary material for: Comparison of rectum fecal bacterial community of finishing bulls fed high-concentrate diets with active dry yeast and yeast culture supplementation
Source: Anim Biosci. 2022 Sep 7;36(1):63–74. doi: 10.5713/ab.22.0215 (PMC9834660; doi:10.5713/ab.22.0215)
Supplement: Supplementary file 2 [file ab-22-0215-suppl2.pdf]

Table S2 Effect of dietary supplementation of active dry yeast and yeast culture on growth performance and blood indexes of finishing bulls (n = 15) [1]

Items†	Treatments‡			SEM	*P*-value
	CON	ADY	YC		
Initial body weight, kg	514	516	513	4.26	0.977
Final body weight, kg	577b	611a	587ab	9.57	0.044
Dietary dry matter intake, kg/day	0.64b	0.99a	0.74ab	0.097	0.044
Average daily gain, kg/day	8.7b	10.4a	9.4ab	0.25	0.015
Feed conversion (feed: gain)	13.5	10.6	12.7	0.59	0.146
TP, g/L	67.83	68.21	67.32	0.28	0.429
TG, mmol/L	0.21c	0.35a	0.29b	0.014	0.001
TC, mmol/L	3.06	2.89	3.01	0.097	0.762
LDL, mmol/L	0.72	0.74	0.7	0.047	0.925
HDL, mmol/L	1.84	1.86	1.81	0.051	0.932
VLDL, mmol/L	0.3	0.28	0.29	0.0064	0.402

†TG, triglyceride; TP, total protein; TC, total cholesterol; LDL, lowdensity lipoprotein; HDL, high-density lipoprotein; VLDL, very lowdensity lipoprotein.

‡CON = control group; ADY = active dry yeast group; YC = yeast culture group.

[a, b, c] Within a row, different letters mean differed significantly ($P < 0.05$).

[1] Geng, C. Y., Ren, L. P., Zhou, Z. M., Chang, Y., & Meng, Q. X. (2016). Comparison of active dry yeast (Saccharomyces cerevisiae) and yeast culture for growth performance, carcass traits, meat quality and blood indexes in finishing bulls. Animal Science Journal, 87(8), 982-988.
