# Supplementary material for: Comparison of rectum fecal bacterial community of finishing bulls fed high-concentrate diets with active dry yeast and yeast culture supplementation
Source: Anim Biosci. 2022 Sep 7;36(1):63–74. doi: 10.5713/ab.22.0215 (PMC9834660; doi:10.5713/ab.22.0215)
Supplement: Supplementary file 3 [file ab-22-0215-suppl3.pdf]

Table S3 Effect of dietary supplementation active dry yeasts (ADY) and yeast cultures (YC) on blood immunoglobulin and blood hormone in finishing bulls (n = 15) [2]

Items†	Treatments‡			SEM	*P*-value
	CON	ADY	YC		
IgA, g/L	0.83	0.77	0.80	0.016	0.409
IgM, g/L	2.63	2.61	2.68	0.045	0.801
IgG, g/L	10.73	10.26	10.31	0.15	0.376
Ghrelin, ng/ml	83.69	101.07**	114.01**	3.27	0.001
Insulin, μIU/ml	16.18	16.91	20.87*	0.66	0.009
Leptin, ng/ml	6.26	6.14	6.21	0.21	0.97
T3, ng/ml	0.98	0.96	1.06	0.029	0.392
T4, ng/ml	83.85	83.36	86.49	0.73	0.199

†T3, triiodothyronine; T4, thyroxine

‡CON = control group; ADY = active dry yeast group; YC = yeast culture group.

Multiple comparison, $*p < 0.05$, $**p < 0.01$.

[2] Geng, C. Y., Ji, S., Jin, Y. H., Li, C. Y., Xia, G. J., Li, Y. M., & Zhang, M. (2018). Comparison of blood immunity, antioxidant capacity and hormone indexes in finishing bulls fed active dry yeast (Saccharomyces cerevisiae) and yeast culture. International Journal of Agriculture and Biology, 20(11), 2561-2568.
