# Supplementary material for: Comparison of rectum fecal bacterial community of finishing bulls fed high-concentrate diets with active dry yeast and yeast culture supplementation
Source: Anim Biosci. 2022 Sep 7;36(1):63–74. doi: 10.5713/ab.22.0215 (PMC9834660; doi:10.5713/ab.22.0215)
Supplement: Supplementary file 4 [file ab-22-0215-suppl4.pdf]

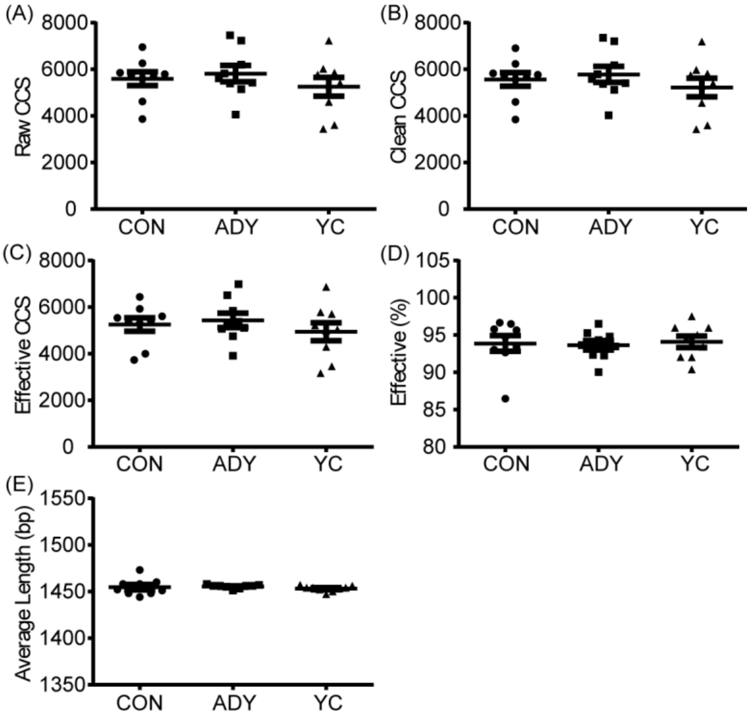

Figure S1. Summary of sequence statistics of all samples. (A) Raw CCS, (B) Clean CCS, (C) Effective CCS, (D) Effective, (E) Average Length. CCS, circular consensus sequences. CON, control group (n = 9); ADY, active dry yeast group (n = 9); YC, yeast culture group (n = 9).
