# Supplementary material for: Comparison of rectum fecal bacterial community of finishing bulls fed high-concentrate diets with active dry yeast and yeast culture supplementation
Source: Anim Biosci. 2022 Sep 7;36(1):63–74. doi: 10.5713/ab.22.0215 (PMC9834660; doi:10.5713/ab.22.0215)
Supplement: Supplementary file 5 [file ab-22-0215-suppl5.pdf]

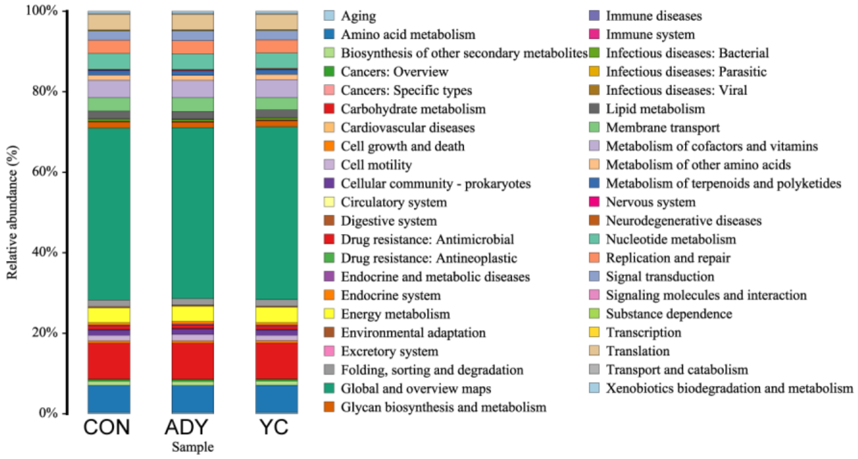

Figure S2. PICRUSt2 prediction results at Class2. CON, control group (n = 9); ADY, active dry yeast group (n = 9); YC, yeast culture group (n = 9).
